# Repellency of zerumbone identified in *Cyperus rotundus* rhizome and other constituents to *Blattella germanica*

**DOI:** 10.1038/s41598-017-16099-6

**Published:** 2017-11-30

**Authors:** Kyu-Sik Chang, Jin-Hwan Jeon, Gi-Hun Kim, Chang-Won Jang, Se-Jin Jeong, Young-Ran Ju, Young-Joon Ahn

**Affiliations:** 1 Division of Vectors and Parasitic Diseases, Centers for Disease Control and Prevention, Osong, 28159 Chungbuk, South Korea; 2 Department of Agricultural Biotechnology, Seoul National University, Seoul, 08826 South Korea

## Abstract

The compounds 1,8-cineole and zerumbone (ZER) from the *Cyperus rotundus* rhizome along with another 11 previously identified rhizome essential oil constituents and α-humulene, which lacks the only carbonyl group present in ZER, as well as binary mixtures of ZER and seven active compounds were tested for repellency to male *B*. *germanica*. The results were compared to *N*,*N*-diethyl-3-methylbenzamide (deet). In filter-paper choice tests, ZER was the most repellent compound, and α-humulene was ineffective, which indicates that the α,β-unsaturated carbonyl group of ZER is a prerequisite component for repellency. At 81.5 μg cm^−2^, enhanced repellency was produced by binary mixtures of ZER and 1,8-cineole, (+)-dihydrocarvone or (*R*)-(+)-limonene (70:30, 50:50 and 30:70 ratios by weight). These mixtures were very effective against male *B*. *germanica* within 24 h and were more repellent than a single compound or deet alone. The optimum ZER content was determined to be more than 50%. In Ebeling choice box tests at 652.4 μg cm^−2^, these compounds and deet resulted in complete repellency to intact male *B*. *germanica*, while they exhibited 35–47% repellency to antennectomized male one. Mixtures formulated from the active constituents of the *C*. *rotundus* rhizome could be useful as potential repellents for controlling *B*. *germanica*.

## Introduction

The German cockroach, *Blattella germanica* L. (Blattodea: Blattellidae), is one of the major sources of potent allergens in sensitive populations^1–3^. Approximately 43% of the United States (US) population (6–59 years old) is allergic to at least one common indoor allergen, and 26% are sensitive to *B*. *germanica* ^4^. Cockroach allergens are associated with feces, saliva, secretions and fragments of their body parts^5^. The prevalence and severity of allergic diseases, such as asthma and rhinitis, caused by cockroaches are increasing, especially among children^1,2^, and the diseases are some of the most serious global public health problems^6–8^. In addition, cockroach exuviae can support large populations of the European house dust mite, which is an important producer of various allergens that lead to exacerbated cases of bronchial asthma^9^.

Cockroach repellents can be used as a preventive tool in the transport and storage of merchandise, protecting sensitive areas such as kitchens, children’s nurseries and hospitals, and reaching inaccessible cockroach hiding places, such as crevices, electrical ducts, plumbing ducts and cabinet voids, as well as for protecting sensitive electronic equipment, communications equipment and food industry facilities^9^. The most widely used insect repellent products are currently based on *N*,*N*-diethyl-3-methylbenzamide (deet)^10^, which continues to be an effective compound. However, deet has many problems, such as having an unpleasant odor and causing damage to certain plastics and synthetic rubber as well as causing medical issues such as central nervous system depression, urticaria and potential encephalopathic toxicity^11^. In addition, the number of approved insecticides and repellents may be reduced in the near future in the US by the US Environmental Protection Agency as re-registration occurs under the 1996 Food Quality and Protection Act^12^. The removal of conventional insecticide or repellent products from markets due to the increase in insecticide resistance or welfare concerns will have a serious impact on the proliferation of *B*. *germanica* ^13,14^. Therefore, there is a pressing need for the development of eco-friendly alternatives for the control of *B*. *germanica*, particularly alternatives with repellency because many insecticides are difficult to apply to inaccessible places, do not reach deep, insecticide-free harborages and cannot be applied to sensitive environments^9^.

Biorepellents and botanical insecticides derived from plants may provide potential alternatives for household and medically important pest insects because plants are a potential source of bioactive secondary metabolites that are perceived by the general public as relatively safe and pose fewer risks to the environment with minimal impacts on human health^15–18^. Many efforts have been focused on plants as potential sources of commercial repellents, in part, because certain plant preparations meet the criteria of minimum-risk repellents^19^. Previous studies have shown that the methanol extract and essential oil from the rhizome of coco-grass, *Cyperus rotundus* L. (Poales: Cyperaceae), possessed good repellency against male *B*. *germanica*. Limited information is available concerning the potential uses of the *C*. *rotundus* rhizome and its constituents for the control of *B*. *germanica*, despite the insecticidal properties of the plant^20^.

In this study, our aim was to assess whether the two monoterpenoids, 1,8-cineole and zerumbone (ZER), which were extracted from *C*. *rotundus* rhizomes, and another 11 previously identified constituents without insecticidal activity from the rhizome essential oil^20^ had repellent activity against male *B*. *germanica* compared to deet, a positive control, in a filter-paper choice bioassay. Deet is registered as an insect repellent in South Korea^21^. The repellency of binary mixtures of ZER and seven active compounds was also evaluated. Finally, the repellency of α-humulene was examined to discern whether the α,β-unsaturated carbonyl group of ZER is essential for repellant activity. The relationship between repellency and boiling points (BPs) of the test compounds is also discussed.

## Results

### Bioassay-guided fractionation and isolation

The fractions obtained from solvent partitioning of the methanol extract of the *C*. *rotundus* rhizome were tested against male *B*. *germanica* using a filter-paper choice assay (Table 1). Significant differences in repellency were observed among the fractions and were used to identify the peak activity fractions for the next step in purification. At a concentration of 244.6 μg cm^−2^, the hexane-soluble fraction was the most potent repellent, while no repellency was obtained using the chloroform-, ethyl acetate- or water-soluble fractions.

**Table 1:** Repellency of fractions obtained from the hydrolyzable solvent of the methanol extract of *Cyperus rotundus* rhizome against male *Blattella germanica* using a filter-paper choice assay at 244.6 μg cm^−2^.

Material	Repellency (%) (±SE)
Methanol extract	100a
Hexane-soluble fraction	100a
Chloroform-soluble fraction	6 ± 1.6b
Ethyl acetate-soluble fraction	8 ± 2.2b
Water-soluble fraction	8 ± 2.4b

Means within a column followed by the same letter are not significantly different (*P* = 0.05, Bonferroni method).

**Figure 1:**
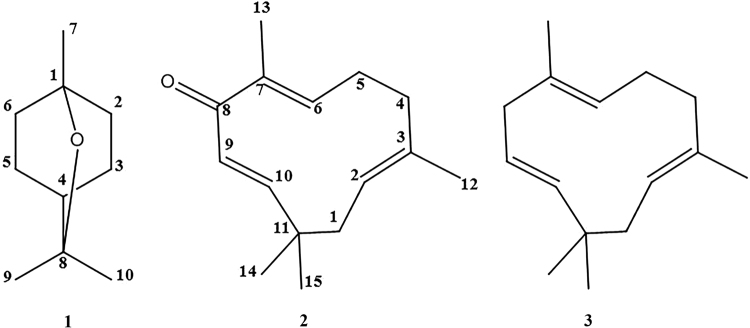
Structures of 1,8-cineole, zerumbone and α-humulene. 1,8-Cineole and zerumbone were identified in the *Cyperus rotundus* rhizome in this study. The chemical formula of 1,8-cineole (**1**) is C_10_H_18_O with a molar mass of 154.249 g/mol. The chemical formula of zerumbone (**2**) is C_15_H_22_O with a molar mass of 218.335 g/mol. α-Humulene (3) is a zerumbone analog that lacks the α,β-unsaturated carbonyl group.

Filter-paper choice bioassay-guided fractionation of the *C*. *rotundus* rhizome led to two active compounds that were identified through spectroscopic analyses, including electron ionized mass spectrometry (EI-MS) and nuclear magnetic resonance (NMR) spectroscopy. The two repellent compounds were 1,8-cineole (**1**) and zerumbone (**2**) (Fig. 1). 1,8-Cineole (**1**) was identified based on the following evidence: colorless oil; EI-MS (70 eV), *m*/*z* (% relative intensity): 154 [M]^+^ (98), 139 (68), 126 (18), 108 (93), 93 (49), 81 (100), 71 (80), 69 (61), 55 (53), 53 (21); ^1^H NMR (CD_3_OD, 400 MHz): δ 1.46–1.95 (m, 4 H), 1.46–1.55 (m, 4 H), 1.33 (m, 1 H), 1.14 (s, 6 H), 0.92 (s, 3 H); ^13^C NMR (CD_3_OD, 100 MHz): δ 75.8 (C-8), 72.0 (C-1), 34.3 (C-4), 32.5 (C-2, C-6), 29.3 (C-9, C-10), 27.8 (C-7), 23.8 (C-3, C-5); and distortionless enhancement by polarization transfer (DEPT) spectra. Zerumbone (**2**) was characterized as follows: pale yellow crystal; EI-MS (70 eV), m/z (% relative intensity): 218 [M]^+^ (85), 203 (9), 189 (10), 175 (7), 163 (21), 150 (28), 135 (100), 121 (17), 107 (85), 96 (61), 91 (30), 67 (24), 53 (23); ^1^H NMR (CD_3_OD, 400 MHz): δ 6.00 (d, *J* = 16.4 Hz, 1 H), 5.86 (s, 2 H), 5.20 (d, *J* = 16.4 Hz, 1 H), 2.40–2.50 (m, 1 H), 2.31–2.40 (m, 1 H), 2.25–2.31 (m, 2 H), 2.15–2.25 (m, 1 H), 1.80 (d, *J* = 13.2 Hz, 1 H), 1.67 (s, 3 H), 1.46 (s, 3 H), 1.11 (s, 3 H), 0.97 (s, 3 H); ^13^C NMR (CD_3_OD, 100 MHz): δ 207.0 (C-8), 163.4 (C-10), 151.4 (C-6), 139.0 (C-7), 137.8 (C-3), 128.2 (C-9), 126.2 (C-2), 43.4 (C-1), 40.5 (C-4), 39.0 (C-11), 29.9 (C-14), 25.6 (C-5), 24.6 (C-15), 15.5 (C-12), 11.9 (C-13); and DEPT spectra. The interpretations of the proton and carbon signals of compounds **1** and **2** were largely consistent with the findings of Liu *et al*.^22^ and Dai *et al*.^23^, respectively.

### Repellency of the tested compounds

The repellency of the 14 compounds and deet, which was used as a positive control, against male *B*. *germanica* was evaluated using a filter-paper choice assay (Table 2). The responses varied according to the compounds and concentrations that were examined. For example, at 163.1 μg cm^−2^, ZER and 1,8-cineole produced complete repellency and the repellency of these compounds decreased considerably with concentration. (+)-Dihydrocarvone, (*R*)-(+)-limonene and (1*S*)-(−)-verbenone resulted in 86, 84 and 83% repellency, respectively. All these compounds were significantly more potent repellents than deet at 163.1 μg cm^−2^. The repellency of (*S*)-(−)-limonene, β-caryophyllene and (−)-(*E*)-pinocarveol did not differ significantly from deet, while the repellency of (−)-caryophyllene oxide was significantly lower than deet. (*E*)-carveol, (−)-α-copaene, α-humulene, (1*R*)-(−)-myrtenal and (1*R*)-(−)-myrtenol were almost ineffective (<5% repellency).

**Table 2:** Repellency of nine pure organic compounds and a commercial repellent, deet, against male *Blattella germanica* using a filter-paper choice assay.

Compound	Repellency, % (±SE), at application rates (μg cm^−2^)			
	326.2	163.1	81.5	16.3
Zerumbone	100 a, A	100 a, A	77 ± 3.2 a, B	31 ± 3.2 a, C
1,8-Cineole	100 a, A	100 a, A	57 ± 3.0 b, B	25 ± 2.5 a, C
(+)-Dihydrocarvone	100 a, A	86 ± 2.2 b, B	57 ± 5.1 b, C	20 ± 3.7 a, D
(*R*)-(+)-Limonene	100 a, A	84 ± 3.7 b, B	34 ± 2.4 c, C	
(1*S*)-(−)-Verbenone	100 a, A	83 ± 2.8 b, B	25 ± 3.9 cd, C	
(*S*)-(−)-Limonene	100 a, A	62 ± 4.9 c, B	19 ± 2.4 de, C	
β-caryophyllene	91 ± 1.5 b, A	54 ± 1.3 c, B	21 ± 2.0 cde, C	
(−)-(*E*)-pinocarveol	89 ± 2.8 b, A	48 ± 4.2 c, B	8 ± 1.6 f, C	
(−)-Caryophyllene oxide	40 ± 2.0 c, A	13 ± 1.7 d, B		
Deet	90 ± 2.8 b, A	53 ± 2.4 c, B	10 ± 2.1 ef, C	2 ± 1.2 b, D

Means followed by the same lowercase letter in the same column or the same uppercase letter in the same row are not significantly different (*P* = 0.05, Bonferroni method).

The repellency of the eight selected active compounds and deet at 652.4 μg cm^−2^ against male *B*. *germanica* was evaluated using Ebeling choice boxes (Table 3). The repellency was significantly different among treatments. ZER resulted in complete repellency at the 10 h post-treatment interval, but after that peak, the cockroach repellency of this compound decreased considerably over time. (−)-(*E*)-Pinocarveol resulted in complete repellency at the 8 h post-treatment interval. (+)-Dihydrocarvone, β-caryophyllene and (1*S*)-(−)-verbenone produced complete repellency at 4 h, while (*R*)-(+)-limonene, (1*S*)-(−)-limonene and 1,8-cineole showed 85–70% repellency. Overall, all these compounds were less potent repellents than deet.

**Table 3:** Residual repellency of eight selected organic pure compounds and the commercial repellent deet against male *Blattella germanica* using an Ebeling choice box assay at 652.4 μg cm^−2^.

Compound^a^	Repellency, % (±SE) at hours after treatment						
	4	8	10	14	16	18	24
Zerumbone	100 a, A	100 a, A	100 a, A	80 ± 8.7 ab, B	60 ± 2.9 b, BC	40 ± 11.5 a, CD	15 ± 2.9 b, D
(−)-(*E*)-pinocarveol	100 a, A	100 a, A	75 ± 8.7 b, B	58 ± 8.8 b, BC	30 ± 7.6 bc, C		
(+)-Dihydrocarvone	100 a, A	90 ± 2.9 b, B	70 ± 5.8 b, BC	50 ± 8.7 b, CD	23 ± 3.3 bc, D		
β-caryophyllene	100 a, A	85 ± 2.9 b, B	75 ± 2.9 b, BC	53 ± 9.3 b, C	20 ± 5.8 c, D		
(1*S*)-(−)-Verbenone	100 a, A	80 ± 7.6 b, AB	75 ± 7.6 b, B	50 ± 11.5 b, BC	20 ± 8.7 c, C		
(*R*)-(+)-limonene	85 ± 2.9 b, A	50 ± 2.9 c, B	17 ± 4.4 c, C				
(*S*)-(−)-limonene	80 ± 5.0 b, A	43 ± 3.3 c, AB	15 ± 7.6 c, B				
1,8-Cineole	70 ± 8.7 b, A	30 ± 5.8 c, B					
Deet	100 a, A	100 a, A	100 a, A	100 a, A	95 ± 2.9 a, A	78 ± 7.3 a, B	48 ± 3.3 a, C

Means followed by the same lowercase letter in the same column or the same uppercase letter in the same row are not significantly different (*P* = 0.05, Bonferroni method).

### Repellency of binary mixtures

The differences in repellency after the application of the seven binary mixtures were significant (Table 4). The repellency of the binary mixtures of ZER and 1,8-cineole, (+)-dihydrocarvone or (*R*)-(+)-limonene at the 70:30, 50:50 and 30:70 ratios was between 94 and 90%, between 91 and 85% and between 82 and 79% at 24 h post-treatment, respectively. The mixtures with the 70:30 ratio were significantly more effective than either the corresponding compound or deet alone. The repellency of the other four binary mixtures at the 70:30, 50:50 and 30:70 ratios was between 83 and 80%, between 79 and 73% and between 73 and 68%, respectively.

**Table 4:** Residual repellency of binary mixtures of zerumbone and seven active compounds against male *Blattella germanica* using a filter-paper choice box assay at 81.5 μg cm^−2^ for 24 h.

Treatment (Zerumbone + compound)	Repellency, % (±SE)					Deet
	Mixture ratio					
	100:0	70:30	50:50	30:70	0:100	
1,8-Cineole	78 ± 0.93 B	94 ± 1.8 A	91 ± 1.7 A	82 ± 1.8 B	68 ± 0.9 C	19 ± 0.7 D
(+)-Dihydrocarvone	76 ± 1.0 C	93 ± 0.8 A	86 ± 1.4 B	80±1.5 C	62 ± 1.0 D	17 ± 0.6 E
(*R*)-(+)-Limonene	73 ± 0.9 C	90 ± 1.5 A	85 ± 2.5 AB	79 ± 2.1 BC	45 ± 1.0 D	14 ± 0.9 E
(*S*)-(−)-Limonene	72 ± 1.0 B	83 ± 2.2 A	76 ± 2.1 AB	72 ± 1.9 B	29 ± 1.2 C	16 ± 1.0 D
(−)-(*E*)-pinocarveol	70 ± 1.0 C	82 ± 2.2 A	79 ± 2.0 AB	73 ± 2.2 BC	16 ± 0.9 D	15 ± 0.7 D
(1*S*)-(−)-Verbenone	73 ± 0.6 B	80 ± 2.1 A	76 ± 2.7 AB	73 ± 2.5 AB	39 ± 1.0 C	15 ± 0.6 D
β-caryophyllene	72 ± 0.7 B	80 ± 1.9 A	73 ± 2.5 AB	68 ± 3.0 B	34 ± 1.1 C	18 ± 0.8 D

Means within a row followed by the same letter are not significantly different (*P* = 0.05, Bonferroni method).

### Behavioral response

Antennectomy of male *B*. *germanica* resulted in a diminished response to the five tested compounds and deet at 652.4 μg cm^−2^ in the Ebeling choice boxes (Table 5). There was a significant difference (*P* < 0.0001) in the repellency of ZER to intact and antennectomized male *B*. *germanica*. Similar differences in the response of male cockroaches to (+)-dihydrocarvone, β-caryophyllene, (1*S*)-(−)-verbenone, 1,8-cineole and deet in intact versus antennectomized treatments were also observed.

**Table 5:** Repellency of five pure organic compounds and commercial repellent deet to antennectomized and intact male *Blattella germanica* using an Ebeling choice box assay at 652.4 μg cm^−2^.

Compound	Repellency, % (±SE)		
	Antennectomized	Intact	*P*-value
Zerumbone	45 ± 2.9	100	<0.0001
(+)-Dihydrocarvone	47 ± 3.3	100	<0.0001
β-caryophyllene	40 ± 2.9	100	<0.0001
(1*S*)-(−)-Verbenone	38 ± 4.4	100	0.0002
1,8-Cineole	35 ± 7.6	100	0.0010
Deet	43 ± 3.3	100	<0.0001

## Discussion

In this study, the repellency of 1,8-cineole and ZER from the *C*. *rotundus* rhizome along with 11 previously identified constituents and binary mixtures of ZER and seven active compounds against male *B*. *germanica* was assessed. The repellency of these materials was compared to deet, which is a currently available synthetic repellent, to determine whether they would be suitable for future commercial cockroach repellents. Historically, *C*. *rotundus* has been widely used as an analgesic, a sedative, an antiplasmodic agent, an antimalarial and a treatment for stomach disorders^24,25^.

Many plant extracts and essential oils produce repellency to different insect species^26–28^. Repellent constituents derived from plants against cockroaches include flavonoids^29^, phenylpropanoids^30^ and terpenoids^31–33^. For example, the monoterpenoids, *E*,*Z*- and *Z*,*E*-nepetalactone as well as pulegol, isopulegol and pulegone work against *B*. *germanica*, and the phenylpropanoids, safrole and isosafrole, work against *Periplaneta americana* (L.) (Blattodea: Blattidae) and have been reported as repellent compounds^30–32^. In the current study, the repellent constituents of the *C*. *rotundus* rhizome were determined to be the sesquiterpenoid ZER and the monoterpenoid 1,8-cineole. Of the tested compounds, ZER was the most potent repellent. Potent repellency was also produced by β-caryophyllene, (+)-dihydrocarvone, (*R*)-(+)-limonene, (*S*)-(−)-limonene, (−)-(*E*)-pinocarveol and (1*S*)-(−)-verbenone. This study is the first report of the repellency of ZER and pinocarveol against *B*. *germanica*, although 1,8-cineole has been reported to have repellency against *B*. *germanica* nymphs^34^ as well as contact and fumigant toxicity and ovicidal and feeding-deterrent activity against *Tribolium castanaeum* (Herbst) (Coleoptera: Tenebrionidae)^35^ and fumigant toxicity against three major stored-grain insects^36^.

Volatile constituents derived from most plant extracts and essential oils consist of alkanes, alcohols, aldehydes and terpenoids (particularly monoterpenoids)^34,37^ and some are insect repellents^38^. Because of their high volatility, the essential oils and their constituents are usually effective against arthropods only for a relatively short period, which is typically less than 2 h^38–40^. However, available information on *B*. *germanica* repellency is limited because most previous studies that investigated repellency used a bioassay with a short time period, which was mainly 5 to 10 min. The compounds 1,8-cineole and α-pinene have been reported to have 92 and 82% repellency, respectively, against *B*. *germanica* nymphs after a 2 h post-treatment at a concentration of 5 ppm^33^. However, Appel *et al*.^41^ reported that mint oil extract at the surface concentration of 2 mL of mint oil to 457.5 cm^2^ was extremely repellent (approximately 100%) to adult males of *B*. *germanica* and *P*. *americana* for 14 days.

In the current study with Ebeling choice box tests, (−)-(*E*)-pinocarveol and ZER were very effective against male *B*. *germanica* for 8–10 h, while (+)-dihydrocarvone, β-caryophyllene and (1*S*)-(−)-verbenone were very effective for 4 h. Nevertheless, these compounds were less potent repellents than deet. The differences in repellency could be attributed to the differences in the quantitative losses because of the different volatility of the compounds, as described by Brown and Hebert^42^. The ability of a chemical vapor to function as a repellent has been reported to be related to its BP, and BPs between 230 and 260 °C at atmospheric pressure were found to be the most desirable for an effective repellent^42^. The BPs of (*E*)-carveol and (−)-α-copaene coincide with the optimal range but were less effective than either ZER with higher BPs, compounds with lower BPs or compounds with similar BPs. Thus, other factors, such as structural characteristics (e.g., types of functional groups and carbon skeleton), rather than BP appear to be associated with repellency of these compounds against male *B*. *germanica*. For example, β-caryophyllene was more effective than caryophyllene oxide. Interestingly, α-humulene, which lacks only the α,β-unsaturated carbonyl group present in ZER, was virtually ineffective. This finding indicates that the α,β-unsaturated carbonyl group of ZER is a prerequisite component for repellency to *B*. *germanica*. Peterson *et al*.^31^ studied the repellency of catnip, *Nepeta cataria* L. (Lamiales: Lamiaceae), as well as its essential oil constituents, against male *B*. *germanica*. They reported that the iridoid monoterpenoid, *E*,*Z*-nepetalactone, was significantly more active than the *Z*,*E*-isomer. In addition, ZER, (+)-dihydrocarvone, β-caryophyllene, (1*S*)-(−)-verbenone, 1,8-cineole and deet produced complete repellency against intact male *B*. *germanica*, while these compounds exhibited 35–47% repellency against antennectomized male *B*. *germanica*. These results indicate that antennae are partly responsible for the perception of the tested compounds. Detailed tests are needed to fully understand their modes of repellent action.

It is well known that repellency against various insect species was more pronounced in binary mixtures of phytochemicals compared to the corresponding single compounds. For example, a significantly enhanced repellency was produced through binary mixtures of *E*,*Z*- and *Z*,*E*-nepetalactone that were consistently more repellent than the isomers when they were tested alone against *Anopheles gambiae* Giles (Diptera: Culicidae), expect for equivalent or near-equivalent mixtures, which had significantly lower repellency^43^. In the current study, repellency against male *B*. *germanica* produced by the binary mixtures of ZER and 1,8-cineole, (+)-dihydrocarvone or (*R*)-(+)-limonene at three mixture ratios were more pronounced compared to repellency produced by the corresponding compound and deet alone. The improved effectiveness of repellency could be attributed to the lower evaporation rate and/or better persistence of ZER in the combined presence of another compound, as described by Khan *et al*.^44^ and Tuetun *et al*.^45^. The optimum ZER content was determined to be more than 50% according to our laboratory results. This original finding indicates that binary mixtures could be promising repellant products that are novel and effective against *B*. *germanica*. However, these terpenoids are volatile, and the volatility problem may be alleviated by special formulations, such as microencapsulation, that can reduce volatile loss, as described by Peterson *et al*.^31^.

In conclusion, *C*. *rotundus* rhizome-derived products containing active compounds, especially zerumbone and 1,8-cineole, could be useful as repellents in the control of *B*. *germanica* populations in sensitive environments in which conventional insecticides would be inappropriate, as long as special formulations (e.g., microencapsulation) that facilitate the slow release of active compounds can be selected or developed. Further research is needed for the practical applications of plant-derived preparations as novel cockroach repellent products to establish their safety profiles in humans. In addition, their effects on non-target organisms and the environment need to be established. Finally, detailed tests are needed to understand how to improve repellency potency and stability for eventual commercial development.

## Methods

### Instrumental analysis

The ^1^H and ^13^C NMR spectra were recorded in CD_3_OD on an Avance 400 WB spectrometer (Bruker, Rheinstetten, Germany) at 400 and 100 MHz, respectively, using tetramethylsilane as an internal standard. The chemical shifts are given in δ (ppm). The DEPT spectra were acquired using Bruker software. The mass spectra were obtained on a GCMS-QP 2010 Ultra gas chromatograph-mass spectrometer (Shimadzu, Columbia, MD, USA). Silica gel 60 (0.063–0.2 mm) (Merck, Darmstadt, Germany) was used for column chromatography. Merck pre-coated silica gel plates (Kieselgel 60 F_254_) were used for analytical thin layer chromatography (TLC). A Spectra System P 2000 high-performance liquid chromatograph (HPLC) (Thermo Scientific, Waltham, MA, USA) was used to isolate the active compounds.

### Chemicals

Two constituents, 1,8-cineole and ZER, were identified in this study, and another 11 previously identified constituents from the *C*. *rotundus* rhizome essential oil^21^ are listed in Table 6 along with their BPs and purities. α-Humulene, which is a zerumbone analog lacking the α,β-unsaturated carbonyl group, was also used in this study for structure-activity relationship (Fig. 1). All compounds were purchased from Sigma-Aldrich (St. Louis, MO, USA). For the relationship between toxicity and BPs of the tested compounds, the BP values of these compounds were obtained from ACD/ChemSketch (ACD/LAB 12.0 for Microsoft Window, Advanced Chemistry Development, Inc., Montreal, Canada) (Table 1). Deet (97.0% purity) was purchased from Sigma-Aldrich. All of the other chemicals used in this study were reagent-grade quality and are available commercially.

**Table 6:** Boiling points of 14 pure organic compounds and commercial repellent deet tested for repellency.

Compound	Boiling point (°C/760 mmHg)	Purity (%)
(*E*)-carveol	231.46	≥95.0
β-caryophyllene	268.36	≥98.5
(−)-Caryophyllene oxide	279.68	95.0
1,8-Cineole	174.01	99.0
(−)-α-copaene	248.50	≥90.0
((+)-Dihydrocarvone	221.50	98.0
α-humulene	276.35	≥98.0
(*R*)-(+)-Limonene	175.44	≥93.0
(*S*)-(−)-Limonene	175.44	≥95.0
(1*R*)-(−)-Myrtenal	215.74	98.0
(1*R*)-(−)-Myrtenol	224.81	95.0
(−)-(*E*)-pinocarveol	217.50	≥96.0
(1*S*)-(−)-Verbenone	227.50	≥93.0
Zerumbone	321.61	≥98.0
Deet	297.45	97.0

### Cockroaches

The stock cultures of *B*. *germanica* (susceptible KSS strain)^20^ were maintained in the laboratory without exposure to any known insecticide. Cockroaches were reared in glass jars (43-cm diameter × 30 cm) containing Purina calf food pellets (Pyeongtaek, Gyeonggi, South Korea), distilled water and a cardboard shelter at 27 ± 2 °C and 60 ± 5% relative humidity (RH) under a 12:12 h light:dark cycle. Because male *B*. *germanica* are more sensitive than females to olfactory stimuli^31,46^, adult males were used for the repellency bioassays.

### Plant material

The rhizome of *C*. *rotundus* was purchased from the Boeun medicinal herb shop (Seoul Yangnyeongsi, Seoul, South Korea). A voucher specimen (CR–01) was deposited in the Research Institute of Agriculture and Life Sciences at Seoul National University.

### Bioassay-guided fractionation and isolation

Air-dried rhizomes (600 g) of *C*. *rotundus* were pulverized, extracted with methanol (3 × 3 L) at room temperature for 2 days and filtered. The combined filtrate was concentrated by rotary evaporation at 40 °C to yield approximately 152 g of a dark greenish tar. The extract (100 g) was sequentially partitioned into hexane- (16.4 g), chloroform- (45.3 g), ethyl acetate- (10.7 g) and water-soluble (27.6 g) portions for the subsequent bioassays. The organic solvent-soluble portions were concentrated under vacuum at 40 °C, and the water-soluble portion was concentrated at 50 °C. To isolate the active constituents, 244.6 μg cm^−2^ of each *C*. *rotundus* rhizome-derived fraction were tested in a filter-paper choice bioassay, as described by Petterson *et al*.^31^.

**Figure 2:**
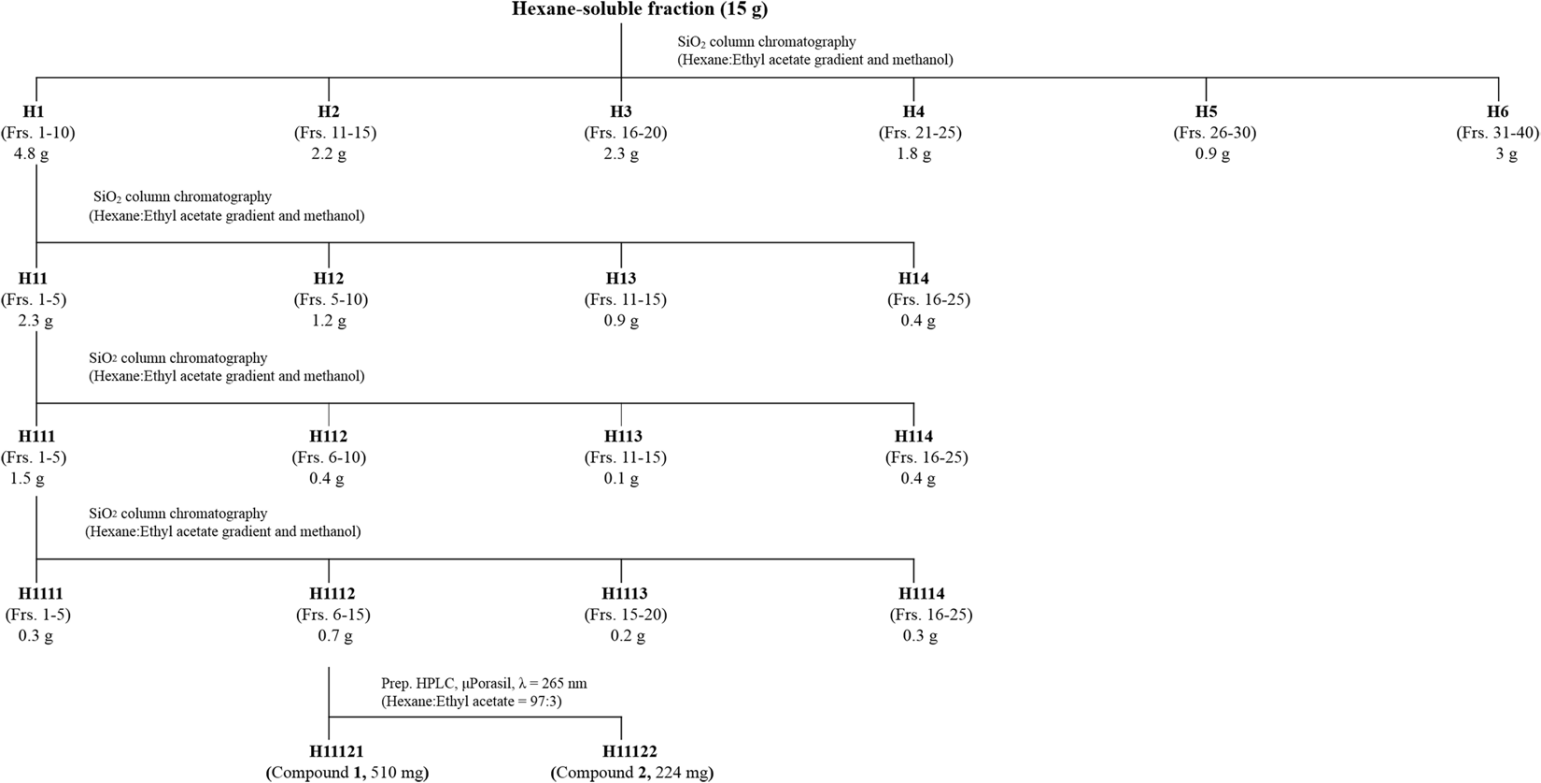
Procedures to isolate the repellent constituents. The *Cyperus rotundus* rhizome methanol extract was sequentially partitioned into hexane-, chloroform-, ethyl acetate- and water-soluble portions. The hexane-soluble fraction was the most biologically active fraction and high-performance liquid chromatography was performed. Each fraction (244.6 μg cm^−2^) was tested in a filter-paper choice bioassay to isolate the active compounds from the fraction.

The hexane-soluble fraction (15 g) was the most biologically active fraction (Table 1) and was chromatographed on a 5.5 × 70 cm silica gel (600 g) column through elution with a gradient of hexane and ethyl acetate [100:0 (2 L), 95:5 (1 L), 90:10 (1 L), 80:20 (1 L) and 70:30 (1 L) by volume] and then elution with methanol (2 L) to provide 40 fractions (each approximately 200 mL) (Fig. 2). The column fractions were monitored by TLC on silica gel plates developed with a hexane and ethyl acetate (7:3 by volume) mobile phase. Fractions with similar *R*_f_ values on the TLC plates were pooled. The spots were detected by spraying the plate with 10% sulfuric acid and then heating the samples on a hot plate. Active fractions 1–10 (H1) were obtained. Fraction H1 was re-chromatographed on a 5.5 × 70 cm silica gel (600 g) column by elution with a gradient of hexane and ethyl acetate [80:20 (1 L), 70:30 (1 L) and 50:50 (1 L) by volume] and a final elution with methanol (2 L) to provide 25 fractions (each approximately 200 mL). Fractions 1–5 (H11) was re-chromatographed on a silica gel column by elution with a gradient of hexane and ethyl acetate [90:10 (1 L), 80:20 (1 L) and 70:30 (1 L) by volume] and a final elution with methanol (2 L) to provide 25 fractions (each approximately 200 mL). Active fractions 1–5 (H111) were obtained. Fraction H111 was re-chromatographed on a silica gel column by elution with a gradient of hexane and ethyl acetate [99:1 (1 L), 90:10 (2 L) and 50:50 (1 L) by volume] and a final elution with methanol (2 L) to provide 25 fractions (each approximately 200 mL). A preparative HPLC was used to separate the constituents from active fractions 6–15 (H1112). The column was a 19 mm i.d. × 300 mm μPorasil (Waters, Milford, MA, USA) with a mobile phase of hexane and ethyl acetate (97:3 by volume) at a flow rate of 2.0 mL min^–1^. Chromatographic separation was monitored using a UV detector at 265 nm. Finally, two active constituents **1** (510 mg) and **2** (224 mg), were isolated at retention times of 10.4 and 13.3 min, respectively.

### Filter-paper choice assay

A filter-paper choice test described previously by Petterson *et al*.^31^ was used to evaluate the repellency of all compounds against male *B*. *germanica* (7–10 days old). Each test was conducted between 20:00 to 23:00 h with overhead florescent lighting at 27 ± 2 °C and 60 ± 5% RH in 5-min test periods, as described previously^31^. A 12.5-cm diameter Whatman no. 2 filter paper (Whatman, Maidstone, UK) was cut in half. One side was treated with different amounts (326.2, 163.1, 81.5 and 16.3 μg cm^−2^) of each test compound in 1 mL methanol, and the other side was treated with 1 mL methanol. Deet served as the positive control and was used in a similar manner. After drying the samples in a fume hood for 1 min, each treated paper was placed on the bottom section of a disposable Petri dish (15-cm diameter × 2 cm). The lid of the dish had a 1 cm hole cut in the center for introducing the cockroach directly into the center of the arena. One insect at a time was introduced. The hole was then blocked by using a small piece of tissue paper to prevent the cockroaches from escaping. Immediately after the introduction of the insect, the number of seconds it spent on the treated or untreated side within 300 s was timed with two stopwatches. If a compound produced ≥40% repellency at a given concentration, further bioassays were conducted. Filter papers and cockroaches were used once then discarded. Each trial was repeated 10 times.

To determine an effective mixture ratio for ZER and another seven active compounds, the repellency of binary mixtures at five tested ratios (100:0, 70:30, 50:50, 30:70 and 0:100 by weight) was tested at a concentration of 81.5 μg cm^−2^ at the 24 h post-treatment, which was based on the preliminary test results. Deet served as the positive control. All treatments were replicated 10 times.

### Ebeling choice box assay

The residual repellency of the eight selected compounds to male *B*. *germanica* was tested in Ebeling choice boxes^47^ as described previously by Appel *et al*.^41^, with a slight modification. A choice test was carried out using a series of two connected acrylic boxes (each 5.5 × 11 × 11 cm). The top side of each compartment of the choice box had nine holes (0.5-mm diameter) on the surface to permit airflow. Except for the top side of the dark compartment of the choice box, the inner surface of other five walls was evenly painted with 652.4 μg cm^−2^ of the test compounds in 2 mL of methanol using a brush, and the amount was based on the preliminary test results. Food and water were placed on the bottom section of the dark compartment of the choice box. Deet served as a positive control and was similarly formulated. Negative controls (i.e., no test material or repellent) consisted of 2 mL methanol only. Treatments were allocated randomly to the choice boxes. After drying the samples in a fume hood for 3 min, 20 adult male *B*. *germanica* were released into the untreated compartment of the choice box and were allowed to enter the treated compartment for 24 h. Cockroaches were able to move freely between the dark (treated) and the lighted (untreated) compartments through two holes (1-cm diameter) in the partition separating the sides. Choice boxes had the same environmental conditions as those used for colony maintenance. Banks of white fluorescent lights were 1.6 m above the choice boxes and produced a light intensity in the untreated compartment of 300–350 lux using an INS Digital Lux Meter (Markson Scientific, Phoenix, AZ, USA). The number of *B*. *germanica* in each compartment was recorded at every 15 min interval until 24 h post-treatment. Six replicates were used for each treatment in a completely randomized design.

For the tests that used antennectomized male *B*. *germanica*, a razor blade was used to remove the antennae at the scape, as described by Petterson *et al*.^31^. The cockroaches were allowed to recover from the procedure for 24 h before they were exposed to 652.4 μg cm^−2^ of each compound and deet in 2 mL methanol according to the choice box method above.

### Data analysis

The repellent index was calculated according to the following formula: % repellency for filter-paper choice assay = [(*Tu* − *Tt*)/*Tn*] × 100, where *Tu* is the number of seconds a cockroach spent on the untreated side, *Tt* is the number of seconds a cockroach spent on the treated side, and *Tn* is the total number of seconds (300)^31^; % repellency for the Ebeling choice box assay = 100 − [(*Ta*/*Tb*) × 100], where *Ta* is the number of cockroaches in the treated group and *Tb* is the number of total cockroaches that were tested^41^. The percentages of repellency were transformed to arcsine square-root values for analysis of variance (ANOVA). The Bonferroni multiple-comparison method was used to test for significant differences among treatments^48^. A Student’s *t*-test was used to test for significant differences between the two treatment methods^48^. A compound with <10% repellency was considered to be ineffective. Means ± standard errors (SEs) of untransformed data are reported.

## Electronic supplementary material

Supplementary Information
