# Supplementary material for: Repellency of zerumbone identified in Cyperus rotundus rhizome and other constituents to Blattella germanica
Source: Sci Rep. 2017 Nov 30;7:16643. doi: 10.1038/s41598-017-16099-6 (PMC5709496; doi:10.1038/s41598-017-16099-6)
Supplement: Supplementary file 1 — Supplementary Information [file 41598_2017_16099_MOESM1_ESM.pdf]

Supplementary information

SREP-16-47776A

Title: Repellency of zerumbone identified in *Cyperus rotundus* rhizome and other constituents to *Blattella germanica*

Authors: Kyu-Sik Chang[1], Jin-Hwan Jeon[1], Gi-Hun Kim[1], Chang-Won Jang[1], Se-Jin Jeong[1], Young-Ran Ju[1] & Young-Joon Ahn[2*]

Figure legend for Supplementary information

Figure S1. EI-MS (70 eV) spectrum of 1,8-cineole 1.

Figure S2. ^{1}H NMR (CD_3OD, 400 MHz) spectrum of 1,8-cineole 1.

Figure S3. ^{13}C NMR (CD_3OD, 100 MHz) spectrum of 1,8-cineole 1.

Figure S4. DEPT spectrum of 1,8-cineole 1.

Figure S5. EI-MS (70 eV) spectrum of zerumbone 2.

Figure S6. ^{1}H NMR (CD_3OD, 400 MHz) spectrum of zerumbone 2.

Figure S7. ^{13}C NMR (CD_3OD, 100 MHz) spectrum of zerumbone 2.

Figure S8. DEPT spectrum of zerumbone 2.

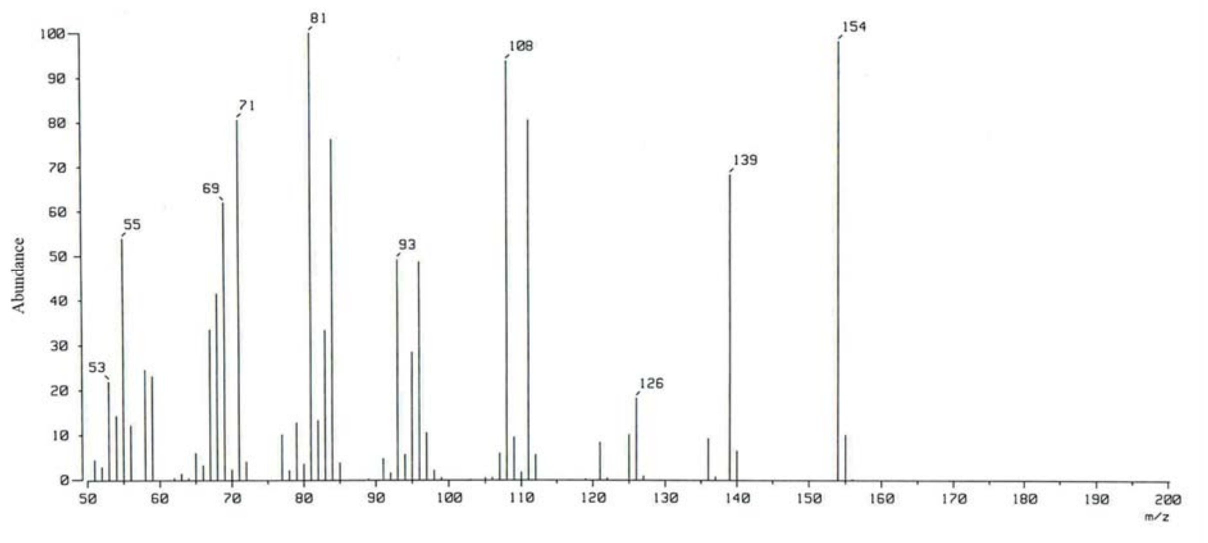

Figure S1.

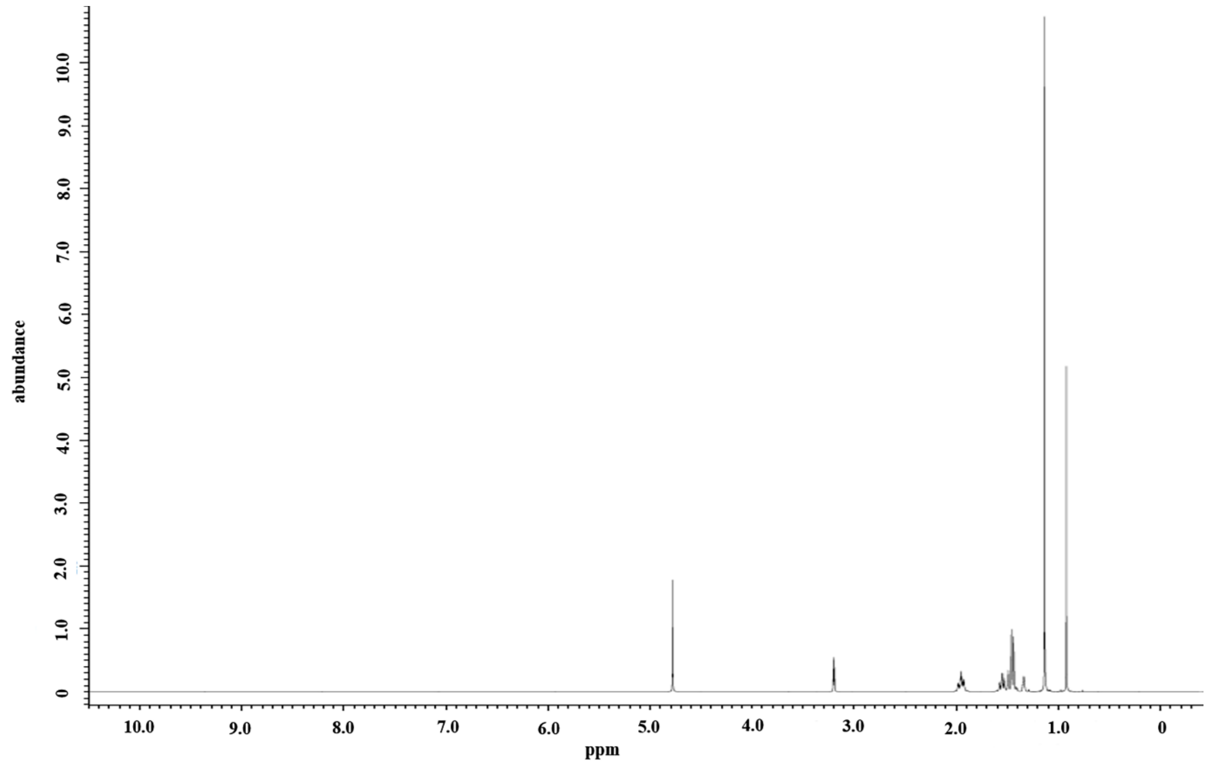

Figure S2.

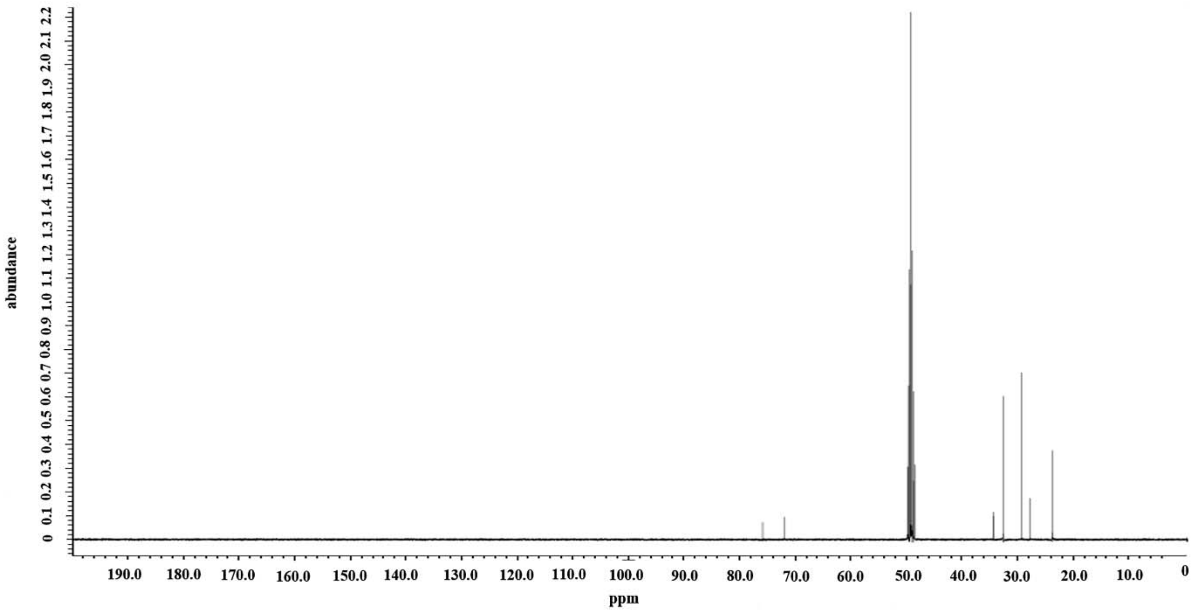

Figure S3.

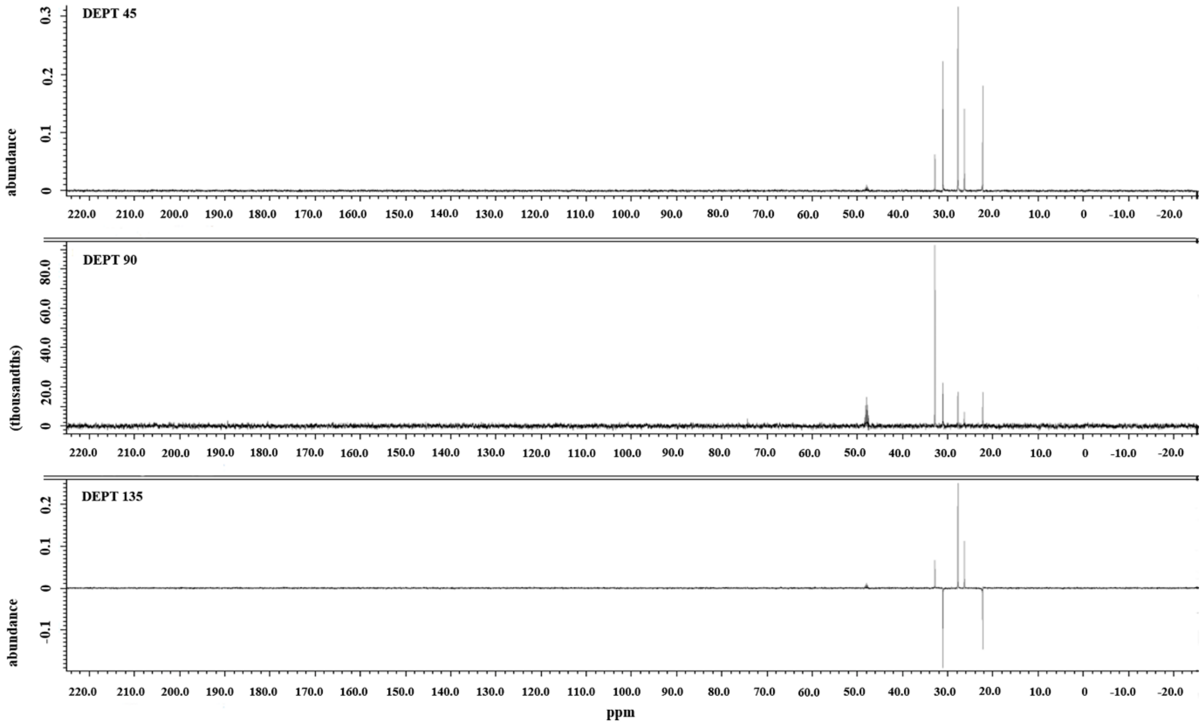

Figure S4.

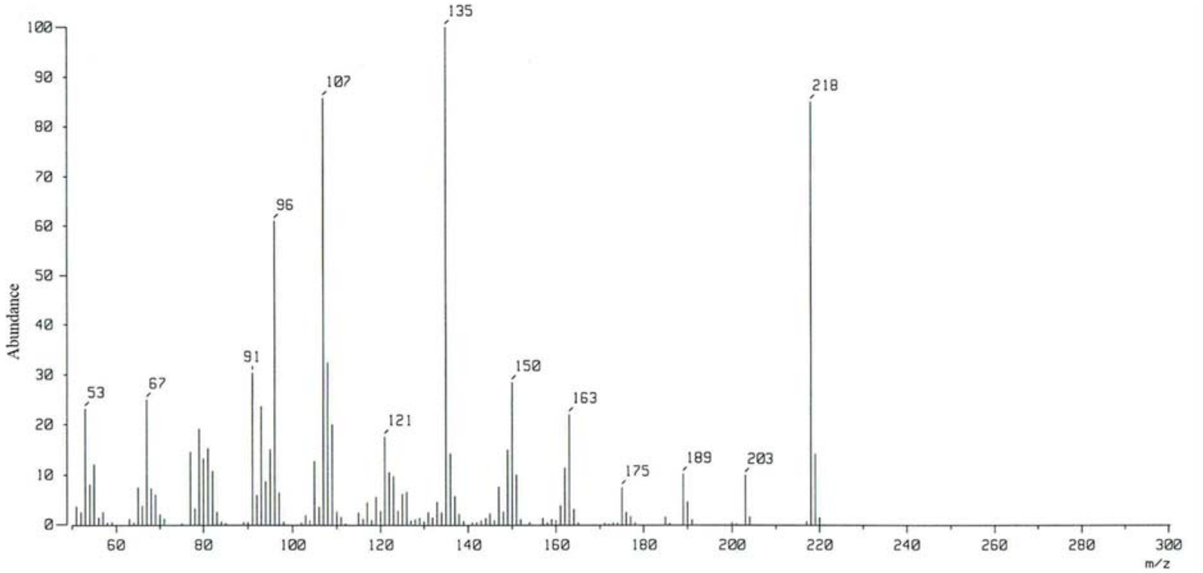

Figure S5.

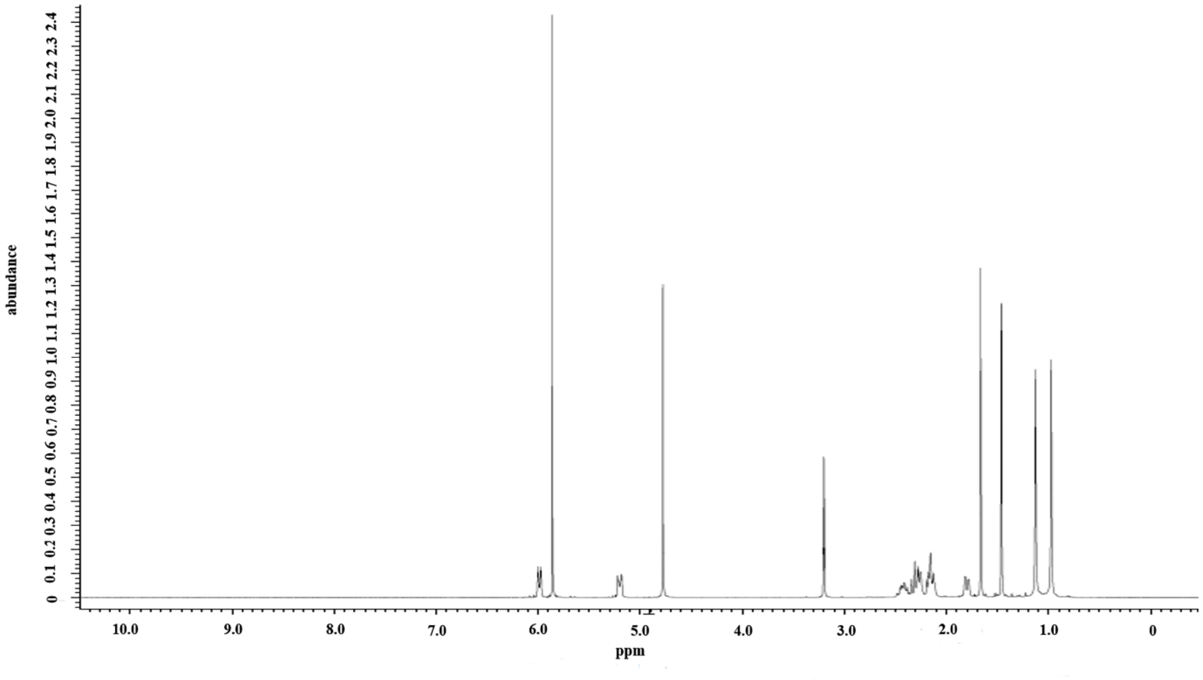

Figure S6.
